# Root plasticity and Pi recycling within plants contribute to low-P tolerance in Tibetan wild barley

**DOI:** 10.1186/s12870-019-1949-x

**Published:** 2019-08-05

**Authors:** Lizhi Long, Xinyi Ma, Lingzhen Ye, Jianbin Zeng, Guang Chen, Guoping Zhang

**Affiliations:** Department of Agronomy, Zhejiang University, Yuhangtang Road 866, Hangzhou, 310058 China

**Keywords:** Phosphorus, Barley, Root, Low-phosphorus tolerance, Pi recycling

## Abstract

**Background:**

Barley is a low phosphorus (P) demand cereal crop. Tibetan wild barley, as a progenitor of cultivated barley, has revealed outstanding ability of tolerance to low-P stress. However, the underlying mechanisms of low-P adaption and the relevant genetic controlling are still unclear.

**Results:**

We identified low-P tolerant barley lines in a doubled-haploid (DH) population derived from an elite Tibetan wild barley accession and a high-yield cultivar. The tolerant lines revealed greater root plasticity in the terms of lateral root length, compared to low-P sensitive lines, in response to low-P stress. By integrating the QTLs associated with root length and root transcriptomic profiling, candidate genes encoding isoflavone reductase, nitrate reductase, nitrate transporter and transcriptional factor MYB were identified. The differentially expressed genes (DEGs) involved the growth of lateral root, Pi transport within cells as well as from roots to shoots contributed to the differences between low-P tolerant line L138 and low-P sensitive lines L73 in their ability of P acquisition and utilization.

**Conclusions:**

The plasticity of root system is an important trait for barley to tolerate low-P stress. The low-P tolerance in the elite DH line derived from a cross of Tibetan wild barley and cultivated barley is characterized by enhanced growth of lateral root and Pi recycling within plants under low-P stress.

**Electronic supplementary material:**

The online version of this article (10.1186/s12870-019-1949-x) contains supplementary material, which is available to authorized users.

## Background

Phosphorus (P) is one of the major essential macronutrients for plant growth and development. The roots of plant acquire P exclusively in the form of inorganic phosphate (Pi) [1]. Despite the fact that total P in soils is relatively abundant, Pi availability is a severe limiting factor for crop productivity due to its low diffusion rate and rapid conversion into organic and inorganic forms, which are not readily available for plant uptake [2]. It is estimated that 43% of the arable land worldwide is deficient in plant-available P [3]. To achieve the optimal yield, considerable amount of P fertilizer should be applied to the soils. However, as P is easily fixed with iron, aluminum and calcium, only 10–25% of the applied P could be used by crops [4]. The P-pool in soils might be the source of diffused pollution, resulting in accumulation of heavy metals and water eutrophication [5]. On the other hand, rock phosphate (phosphorite) used as crucial resource for P fertilizer production is non-renewable and being exhausted rapidly. Thus, understanding the mechanisms of Pi-uptake/utilization and screening P-efficient crop genotypes are quite imperative for developing the cultivars with high P use efficiency.

In plants, the concentration of P ranges from 0.05 to 0.50% on the base of dry weight, and Pi is approximately 5 to 20 mM [6]. P functions as a component of macromolecules, such as nucleic acids, proteins and phospholipids, and plays a fundamental role in numerous biological processes, such as energy transfer and metabolism, photosynthesis, respiration, enzyme catalysis and nitrogen fixation [7, 8]. In soils, the concentration of soluble P seldom exceeds 10 μM and is much lower in the rhizosphere [9]. Toward to poor Pi availability in soils, plants have evolved well-regulated systems for Pi acquisition and recycling, including the high- and low-affinity Pi transporters and the ability to induce root architectural changes to forage P [10]. The adjustments of numerous cellular processes are triggered by P starvation and regulated by various factors, including transcriptional factors, microRNAs, phytohormone and sucrose.

Root active transport systems with high-affinity are required for Pi uptake, which is mediated by Pi/H^+^ symporters belonging to the PHT1 (Phosphate transporter 1) family [9]. PHT1, located to plasma membrane, is mainly expressed in roots and functions in Pi uptake and mobilization within plants [11]. Knock-down of PHT1 genes significantly decreased the Pi uptake from soils and the long-distance transport of Pi from roots to shoots [12]. The members of PHT1 were identified in many species, for instance, 9 in *Arabidopsis*, 13 in rice, 11 in barley and 6 in maize. Different from PHT1, PHT2/3/4 are mainly localized to plastids, mitochondria and other organelles, controlling Pi transport activities and being crucial for maintaining Pi homeostasis [13–15].

After taken up by roots, most P would be transported into shoots. PHO1 (Phosphate1), one endomembrane transporter of SPX-EXS(SYG1/PHO81/XPR1-ERD1/XPR1/SYG1) subfamily, is responsible for Pi efflux out of cells into xylem vessel and thereby involved in Pi transport from roots to shoots [16, 17]. The membrane-spanning EXS domain of *AtPHO1* is required for Pi export and proper localization to the Golgi and trans-Golgi network (TGN) [18]. Moreover, SPX-MFS (Major Facilitator Superfamily) is mainly localized to the tonoplast. Three rice proteins of SPX-MFS are predicted to facilitate the transport of Pi across tonoplast and responsible for Pi homeostasis in vacuole [19, 20]. OsSPX-MFS1 and OsSPX-MFS2 are the targets of low-P induced miR827 and suppressed by Pi starvation [19, 21]. In Pi-depleted tissues, miR399 as a systemic signal moving from shoots to roots, is specifically and highly induced [22, 23]. Like a bridge, *PHO2* is suppressed by miR399 post-transcriptionally and regulates PHT1 abundance post-translationally [24, 25]. In addition, PHF1 (Phosphate transporter traffic facilitator1) can also mediate PHT1 protein by controlling its proper trafficking and targeting to plasma membranes [26]. Besides, transcripts of *PHT1* are positively regulated by PHR1, WRKY and sugars, and negatively mediated by cytokinin, abscisic acid, MYB62, ZAT6, SPX3, and ARP6/H2A.Z [27]. Although the relevant genes involved in Pi- starvation response have been reported in model plants, the underlying precise regulatory mechanisms for low-P tolerance in crops remain elusive.

To develop P-efficient crop cultivars and to understand the underlying mechanisms, numerous QTLs associated with P efficiency have been identified in many crops, including maize, wheat and oilseed rape [28–30]. However, few causal genes have been identified up to date, because of the fact that P efficiency in plants is a quantitative trait controlled by minor multiple genes and interacted strongly with the environments. On the other hand, the poor mobility and temporal heterogeneity of plant-available P in soils highlight the importance of root traits for Pi acquisition. Therefore, plant ideotype of crops, i.e. genotypes with reduced metabolic cost of soil exploration, were proposed for improving Pi acquisition [31]. Indeed, the crops with more biomass allocation to roots could increase P capture from low P soil and sustain optimal shoot growth. Thus, the adaption of roots to low-P stress should be given priority in consideration of selective parameters related to P efficiency. On the other hand, the construction of multi-genetic populations and mapping are costly and time-consuming. Therefore, integrating QTL mapping and transcriptomic analyses might be an efficient approach for identifying candidate genes.

Modern crop breeding programs have dramatically narrowed the genetic diversity in cultivated plants and minimized their tolerance to environmental stresses [32]. Barely (*Hordeum vulgare*) is one of the most important cereal crops worldwide, displaying a wide adaption to extreme environments. Tibetan wild barley (*Hordeum spontaneum*), as a progenitor of cultivated barley, has been thought as a unique genetic pool containing many rare elite alleles [33]. The wild barley accessions have been reported with outstanding abilities of tolerance to various abiotic stresses, including drought, salinity, aluminum toxicity and nitrogen deficiency [34–37]. Low-P tolerant accessions were identified in the previous study [38], however, the molecular mechanisms and the relevant genes are not clarified.

In the present study, a DH population from a cross of X26 (low-P tolerant wild barley) and ZD9 (high-yield barley cultivar) was used to investigate the change of root growth in response to low-P stress, and to identify QTLs related to low-P response. The DH population lines were further screened with aims at revealing the mechanisms of low-P tolerance and identifying the relevant candidate genes by integrating QTL mapping and transcriptomic analyses.

## Results

### Phenotypic variation among the lines of X26/ZD9 DH population

**Fig. 1:**
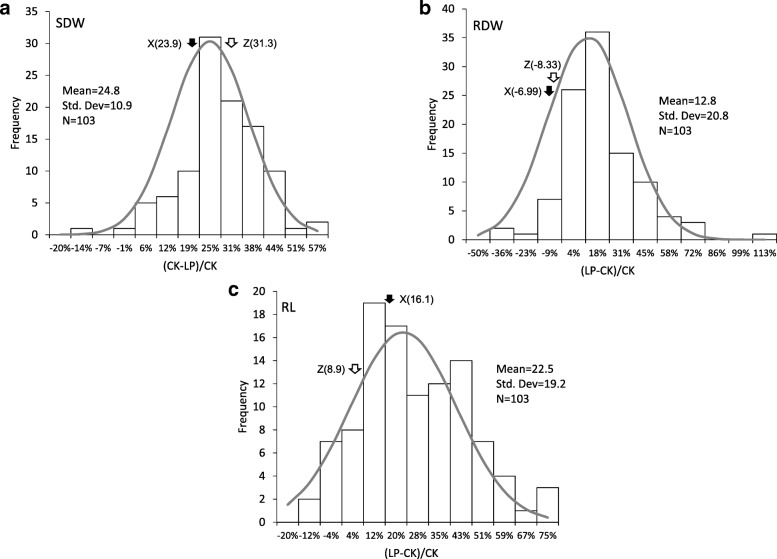
Frequency distribution of the traits of ZD9/X26 DH population grown in low-P condition for three weeks. **a** Shoot dry weight (SDW), **b** root dry weight (RDW) and (**c**) root length (RL) of the 103 DH lines with four to five replicates for each trait

The shoot dry weight (SDW), root dry weight (RDW) and root length (RL) were evaluated on the plants exposed to low-P treatment and control at seedling stage. Compared to the control, the SDW (RDW) of X26 and ZD9 under low-P condition decreased by 23.9% (6.99%) and 31.3% (8.33%) (Fig. 1a, b), respectively. In contrast, the RL of X26 and ZD9 increased by 16.1 and 8.9% under low-P, respectively (Fig. 1c). Among the DH lines, a great variation in the relative SDW, RDW and RL was observed (Fig. 1). In comparison with control plants, SDW and RDW in the low-P treated plants ranged from − 16.8 to 53.9% and − 43.3 to 105.8%, with a mean of 24.8 and 12.8%, respectively. Relative RL ranged from − 16.04 to 71.09% with a mean of 22.5%.

### Growth response of DH lines to low-P stress

**Fig. 2:**
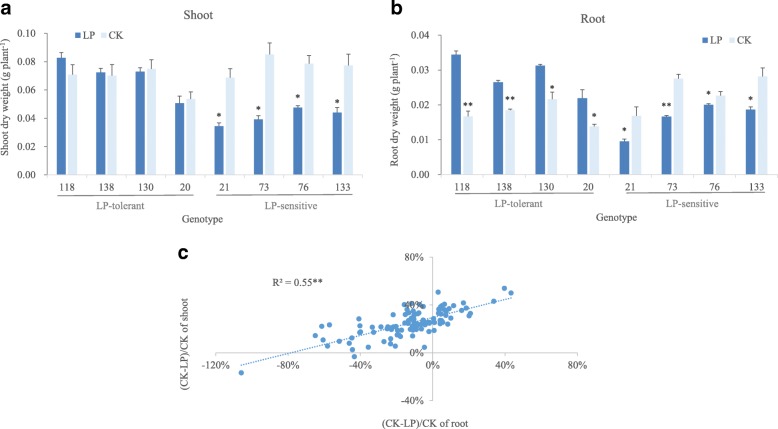
Contrastive response of shoot biomass and root biomass to low-P stress in DH lines. **a** Shoot and (**b**) root biomass of representative lines selected from DH population under low-P stress. **c** Correlation of shoot and root in response to low-P stress in DH population

In order to screen the lines with the extreme response to low P, shoot and root biomass was used as the selective parameter. Under low-P stress, approximately 70% of the DH population lines showed an increase of root growth, compared with the control, and about 30% of the lines showed the reduction of shoot and root biomass. Accordingly, four most tolerant and four most sensitive lines in response to low P were selected (Fig. 2). Lines 118, 138, 130 and 20 were thought as low-P tolerant, as no significant difference was found in their SDW between low-P treatment and control. Whereas, lines 21, 73, 133, 145 were thought as low-P sensitive, as they showed a dramatic reduction of SDW under low-P treatment. Interestingly, root growth was enhanced in the tolerant lines and reduced in the sensitive lines under low-P stress in comparison with control.

**Fig. 3:**
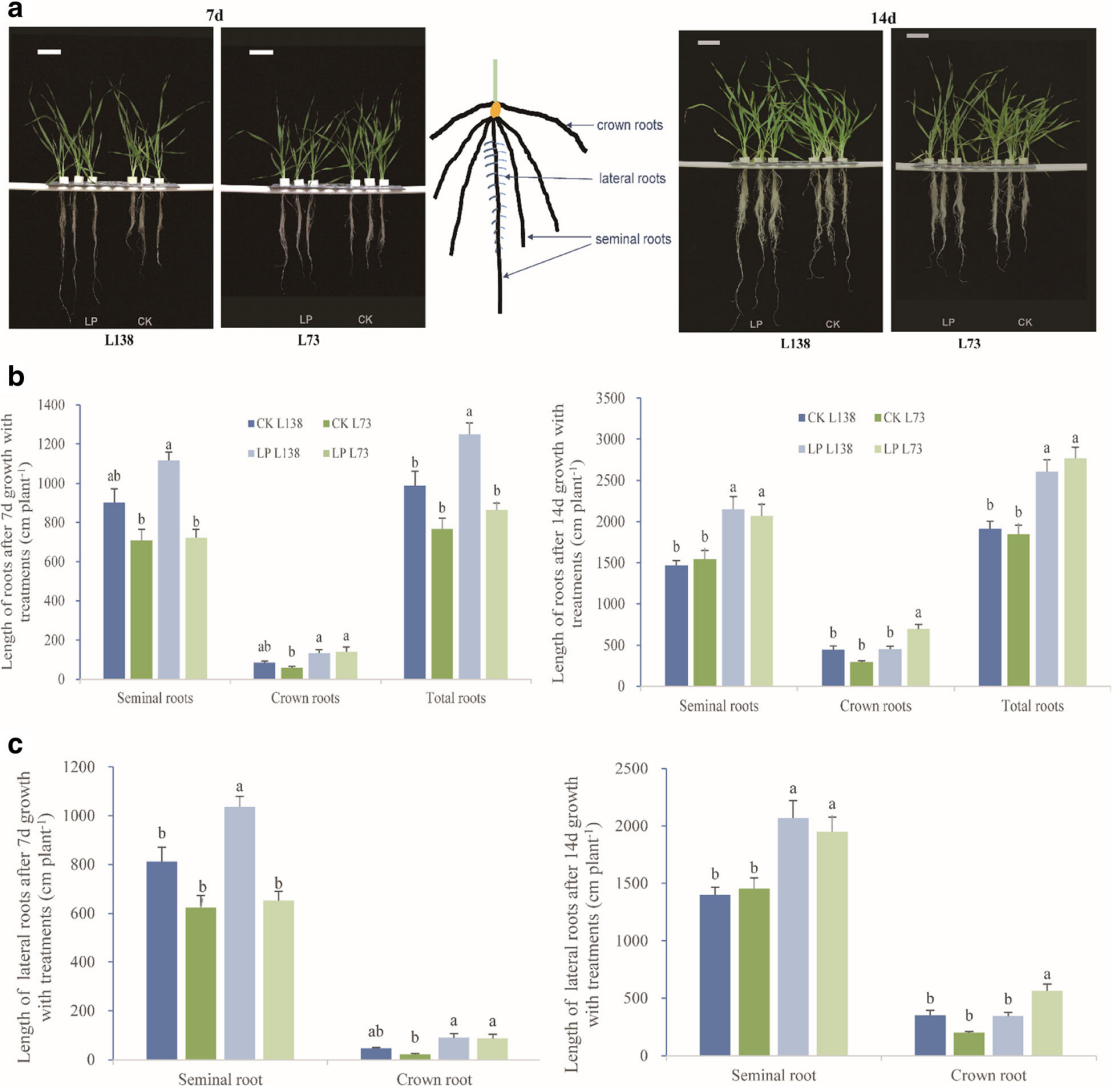
Effects of low-P stress on the root growth of two barley lines with different low-P response. **a** Representative photos of L138 (low-P tolerant) and L73 (low-P sensitive) grown in low-P and control conditions for 7 and 14 days. **b** Total length and (**c**) lateral root length of seminal and crown roots. Different root types are sketched in (**a**). Data are means ± SE (*n* = 6), and different letters indicate significant differences (*p* ≤ 0.05) among columns in the same group

Furthermore, L138 and L73 were used as the lines with extreme low-P response for further physiological and transcriptomic analyses. After 7 days under low-P treatment, tissue P concentration was greatly reduced in both lines, but there was no significant difference in shoot biomass between low-P treatment and control (Table 1). Moreover, the tolerant line L138 was increased in root biomass, whereas the sensitive line L73 had little change under low-P stress (Table 1, Fig. 3a). At 14 day after low-P treatment, the two lines showed the significant reduction in shoot biomass and increase in root biomass (Table 1, Fig. 3a). SDW of L138 and L73 reduced by 13.5 and 31.8%, respectively. While the RDW of L138 increased by 96.8%, being much more than that of L73 (28.2%). Moreover, there was no significant difference in shoot and root biomass between the two lines when grown under control condition.

**Table 1:** Biomass, P concentration and accumulation of barley L138 (low-P-tolerant) and L73 (low-P-sensitive) under low P and control conditions after 7 and 14 days

Treatment	Genotype	Biomass (g DW plant^− 1^)			P concentration (mg P g^− 1^ DW)		P accumulation (mg P organ^− 1^)		Ratio of P in root to total (%)
		Root	Shoot	R:S	Root	Shoot	Root	Shoot	
7d									
Control	L138	0.040 ± 0.001b	0.119 ± 0.006a	0.351 ± 0.028b	9.06 ± 0.14b	11.46 ± 0.18a	0.72 ± 0.04a	1.21 ± 0.10a	0.38 ± 0.03a
	L73	0.039 ± 0.003b	0.099 ± 0.005ab	0.397 ± 0.045ab	**10.08 ± 0.21a**	10.63 ± 0.52a	0.78 ± 0.12a	1.23 ± 0.17a	0.39 ± 0.01a
Low P	L138	0.053 ± 0.001a	**0.113 ± 0.005a**	0.466 ± 0.043ab	2.20 ± 0.05c	2.72 ± 0.27b	0.23 ± 0.00b	0.33 ± 0.01b	0.42 ± 0.01a
	L73	0.046 ± 0.003ab	0.079 ± 0.006b	0.551 ± 0.041a	1.79 ± 0.01c	2.31 ± 0.20b	0.17 ± 0.01b	0.21 ± 0.03b	0.44 ± 0.05a
14d									
Control	L138	0.063 ± 0.003c	0.258 ± 0.011a	0.251 ± 0.012b	9.39 ± 0.04b	**10.78 ± 0.21a**	1.19 ± 0.09b	**3.01 ± 0.14a**	0.28 ± 0.01c
	L73	0.071 ± 0.002c	0.245 ± 0.009ab	0.292 ± 0.021b	**10.81 ± 0.18a**	9.76 ± 0.14b	**1.53 ± 0.06a**	2.23 ± 0.19b	**0.41 ± 0.03b**
Low P	L138	**0.124 ± 0.007a**	**0.223 ± 0.008b**	0.568 ± 0.040a	1.50 ± 0.05c	1.31 ± 0.07c	0.39 ± 0.04c	0.28 ± 0.05c	0.59 ± 0.04a
	L73	0.091 ± 0.002b	0.167 ± 0.004c	0.536 ± 0.018a	1.36 ± 0.03c	1.39 ± 0.01c	0.24 ± 0.01c	0.23 ± 0.00c	0.51 ± 0.01ab

Barley plants were grown in hydroponics under low P and control conditions for 7 and 14 days. Data are mean values ± SE (*n* = 3–6), different letters show significant differences (*p* ≤ 0.05). Bold numbers indicate significant differences between two lines under control or low-P conditions

In view of the great difference between L138 and L73 in root growth under low-P stress, root morphology was further analyzed (Fig. 3b). Exposed to shorter P deficiency (7 days), the total length of roots increased significantly in L138, whereas had no significant change in L73. Similarly, the length of seminal and lateral roots also increased in L138 but not in L73 (Fig. 3c). As the seminal root plays the crucial role in P uptake at seedling stage, the enhancement of its growth under low-P stress is obviously beneficial for L138 to get more P.

Although there was no significant difference in P distribution of the two lines between low-P treatment and control at 7 day after treatment, P distribution into root increased by about 100% for L138 and 24% for L73 under low-P in comparison with control at 14 day after treatment. In addition, L138 and L73 showed no significant difference in shoot P concentration but significant difference in shoot biomass under low-P stress.

### Transcriptomes of the two DH lines under low-P condition

**Fig. 4:**
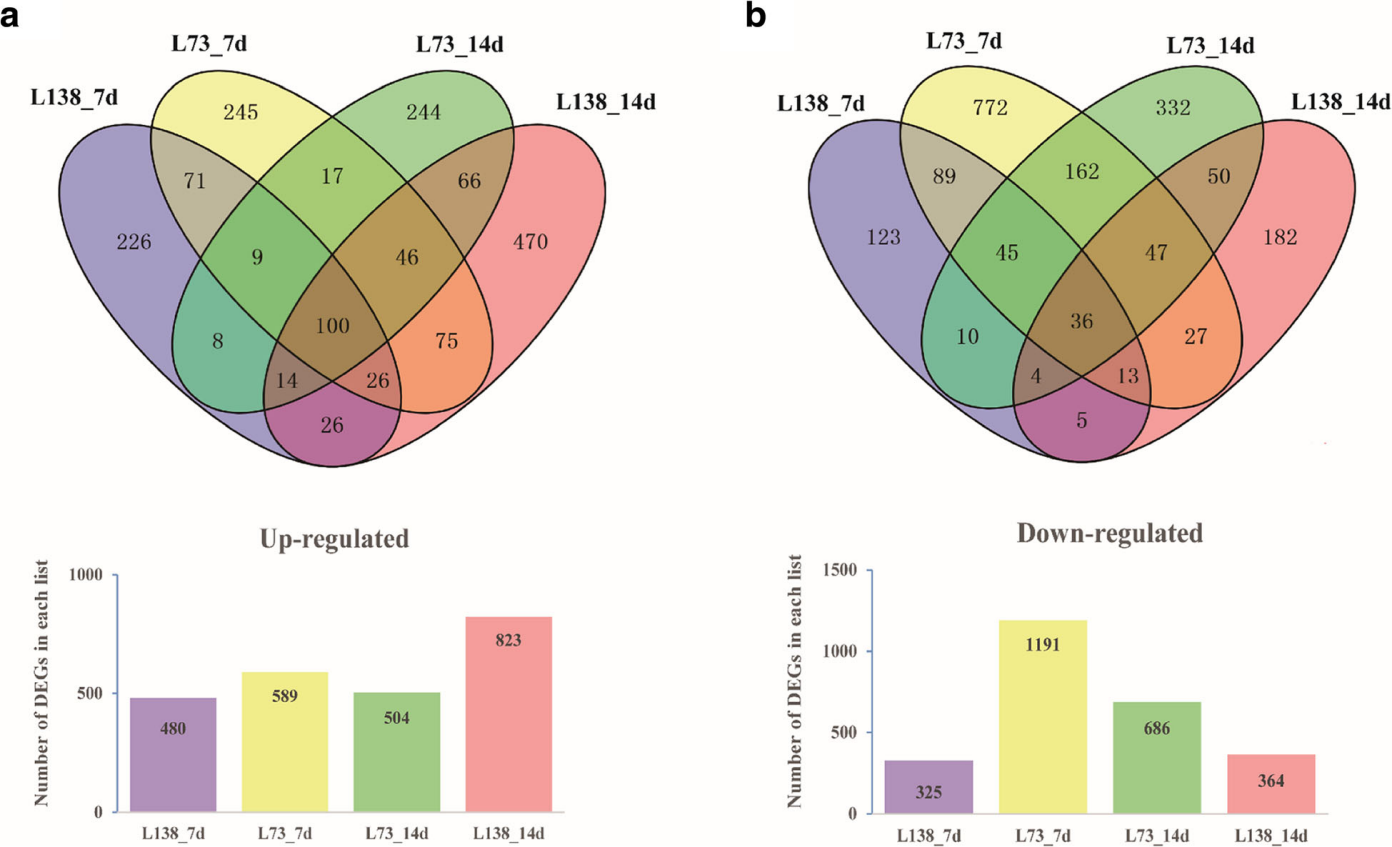
Venn diagram representing the overlaps of phosphate starvation response genes in L138 and L73. **a** Up-regulated genes at 7d and 14d by low-P treatment. **b** Down-regulated genes at 7d and 14d by low-P treatment

To obtain the transcriptomic profiling of the two extreme lines responding to low-P stress, 24 cDNA libraries were constructed with three biological replicates for each sample and thereby sequenced using Illumina HiSeq platform. A total of 10,933,411 clean reads were detected in all examined samples and approximately 70% of the clean reads were mapped to barley genome, yielding 25,559 expressing genes on an average for each sample. The transcriptional levels were normalized by employing FPKM (fragments per kilo-base of exon per million fragments mapped reads) method. Differentially expressed genes (DEGs) were identified according to the fold change and *P* value [39]. The threshold was set as fold change ≥2 at least in one sample and *P* ≤ 0.05 for DEGs. Consequently, 1761 DEGs in L138 and 2493 DEGs in L73 were identified using pairwise comparison between low-P and control, with 789 DEGs being shared by the two lines (Fig. 4). Under low-P stress, 415 genes were up-regulated, and 321 genes were down-regulated in both L138 and L73. At 7 and 14 days after low-P treatment, the number of DEGs was 805 and 1187 for L138, while for L73, the corresponding value was 1780 and 1190, respectively.

To validate the RNA-Seq data, five responsive genes were selected for real-time qPCR analyses, including PHO1-3 (HORVU7Hr1G047210), ARF2 (HORVU3Hr1G096510), SPX-MFS1 (HORVU2Hr1G094690), SPX-MFS2 (HORVU6Hr1G065710) and sugar transporter7 (HORVU2Hr1G079720). The results were consistent with those from RNA-Seq analyses (Additional file 1: Figure S1). Totally 766 DEGs were specially addressed because of their great difference (more than four folds) between L138 and L73 (Additional file 2: Figure S2a). The analyses of their functional categories revealed that more than 50% DEGs were involved in the processes of RNA, protein, transport, stress, signaling and hormone metabolism (Additional file 2: Figure S2b).

**Fig. 5:**
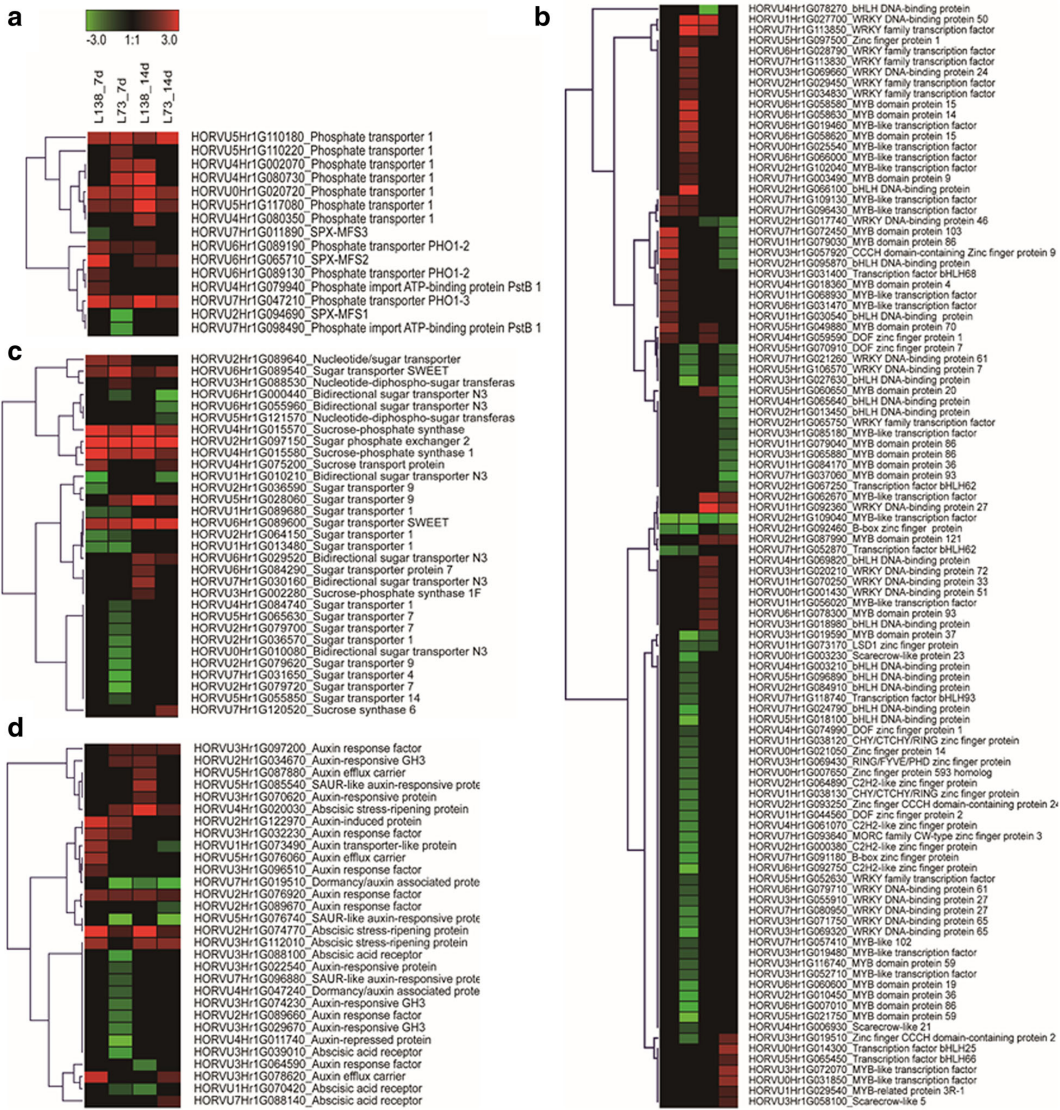
Hierarchical cluster analyses of phosphate starvation response genes identified in differentially expressed genes (DEGs). **a** Phosphate transporters, **b** transcription factors, (**c**) sugar transport related gene, (**d**) auxin and abscisic acid signal pathways. The genotypes and treatments are displayed above each column. DEGs are displayed by different colors. Relative levels of expression are shown by a color gradient from low (green) to high (red)

In the present study, the identified DEGs responding to low-P stress could be categorized into phosphate transporters, transcriptional factors, sugar transport related and hormone signaling. The proteins of PHT1 family, localized to the plasma membranes, are well known for their roles in Pi uptake from soil. Under shorter exposure to P deficiency, more genes in *PHT1* family were up-regulated in L73 than in L138 (Fig. 5a), while at later stage fewer *PHT1* genes were up-regulated in L73 than L138. Furthermore, the proteins of PHO1 involved in Pi loading into xylem and Pi transport from roots to shoots were up-regulated under low-P stress in both lines, but the changed folds of these proteins were higher in L138 than in L73. Besides, more genes related to inner Pi transport including SPX-MFSs and PstB1were up-regulated in L138 compared with L73.

Totally, 126 DEGs encoding transcriptional factors (TF) were identified in response to low-P stress, and they belonged to five families, including MYB (43), WRKY(26), bHLH(30), Zinc finger(14) and Scarecrow-like (3) families (Fig. 5b). Most of these TFs were up-regulated in L138, and more than 60% of TFs were down-regulated in L73, indicating the different expression and regulation pattern between the two lines responding to P starvation. On the other hand, the role of sucrose transport in phloem has been well known in integrating root response to low-P stress, and sugar transport was considered vital for long-distance signaling and maintaining Pi homeostasis. In total, we detected 31 DEGs involved in sucrose/sugar transport and synthase in the two lines (Fig. 5c). Among them, 13 DEGs were commonly found in both lines, and other DEGs were exclusively detected in one line. Nine of them were down-regulated and only expressed in L73. Similarly, among the 30 DEGs involved in auxin/abscisic acid response, 14 DEGs in L73 were down-regulated and 15 DEGs in L138 were up-regulated (Fig. 5d). It has been reported that auxin is involved in the response of lateral roots to P starvation [40]. The different expression of sugar/auxin-related DEGs between the two lines could account for their difference in root length.

In addition, a cluster with 25 DEGs encoding the proteins of NRT1/ PTR (NPF) family was identified (Additional file 3: Figure S3). Among the L73-specific DEGs (12), 10 DEGs were down-regulated, and 4 of 5 L138-specific DEGs were up-regulated under low-P stress. In view of great effect of nitrate on lateral root growth, it may be suggested that the different expression of NRT1/ PTR family in the two lines contributes to their difference of root length.

### Co-localization of DEGs in QTL region

One QTL located on chromosome 6H was associated with the RL change in response to low-P and could account for 9.0% of the phenotypic variance. Although the QTLs associated with root diameter, root volume and grain yield under P treatment were reported in chromosome 6H [41, 42], all of them are located in the different regions as the one we found in the present study.

**Fig. 6:**
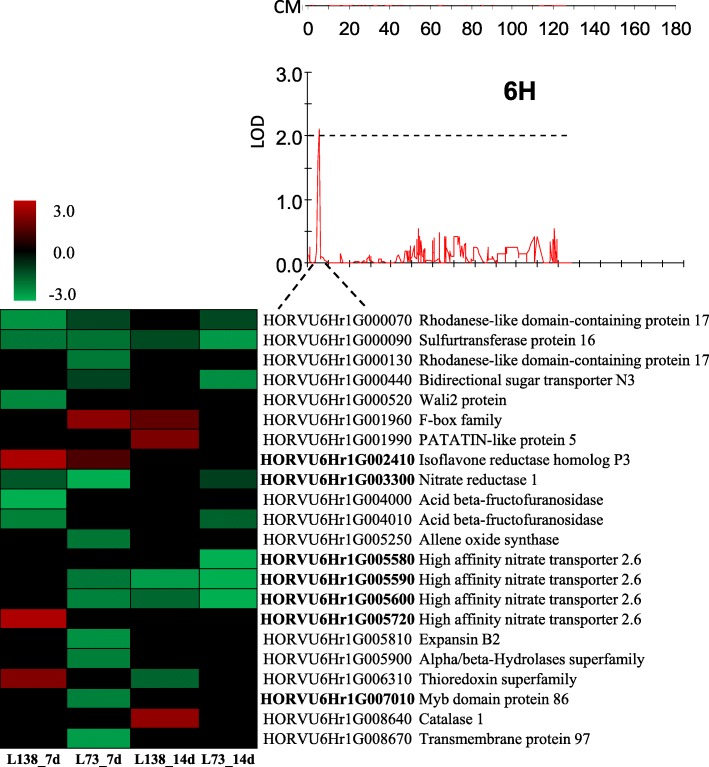
Potential gene candidates detected by RNA-seq underlying QTL regions. The red and green indicate increased and decreased expression of genes, respectively, in L138 and L73 in response to low-P stress

To obtain the candidate genes associated with the identified QTLs, low P-responsive genes with difference of expression (fold change ≥2) between two genotypes were screened in the target region. Consequently, 22 candidate genes in roots were identified in the QTL region (Fig. 6). The proteins encoded by these genes included sugar transporter, isoflavone reductase, nitrate reductase, expansin B, MYB domain protein and a cluster of high affinity nitrate transporters. Specially, one of the four high affinity nitrate transporters was only expressed in L138 and up-regulated under shorter exposure to P starvation (7 days), while the other three were down-regulated. In addition, *HORVU6Hr1G007010* encoding MYB domain protein was only expressed in L73 and down-regulated in response to low P stress.

## Discussion

P is indispensable for plant growth and development, and its deficiency will cause severe reduction of crop yield. The cultivars with high-P use efficiency or low-P tolerance are required to ensure the stable yield under low-P condition or to reduce P fertilizer application. Tibetan wild barley has been proved to be rich in the elite genotypes adapting well to the harsh environments. In the present study, a DH population from a cross of Tibetan wild barley X26 (low-P tolerant) and cultivated barley ZD9 (high yield) was used for investigating the response to low P stress (P starvation) and QTL mapping. The two lines showing the significant difference in the response to low-P were further used for transcriptomic profiling.

### Root plasticity and low-P tolerance

It has been well-documented that plants could alter the distribution of assimilates in response to shorter P-deprivation and thereby lead to an increase in root exploratory capacity [31]. Under longer exposure to low-P stress, plant photosynthesis was greatly inhibited, causing reduced shoot and root biomass [8]. In this study, almost all the DH population lines showed the reduction of SDW under low-P stress. While about two thirds of the DH lines were increased in root growth under low-P stress, indicating the distinct difference in response of root growth to low-P stress among the DH population lines. Among the lines with increased root growth under low-P stress, the differences in shoot biomass change were also observed, and the reduction of SDW was significantly correlated with the change of RDW (Fig. 2c). Furthermore, we found the low-P tolerant L138 had faster and more increase in root length, mainly caused by lateral root growth, in comparison with low-P sensitive L73 under low-P stress. It may be suggested that the plasticity of root system contribute to the differences among barley genotypes in their tolerance to low-P stress.

The previous studies on *Arabidopsis*, rice, maize and oilseed rape highlighted an important role of root architectural plasticity in P acquisition [43–46]. However, a few causal genes have been identified by using QTL mapping. In rice, a cluster of QTLs controlling root growth were detected to be related with P-efficiency [47]. A major QTL was subsequently named as *Pup1* [48]. By narrowing the *Pup1* candidate region and utilizing unique molecular markers, a protein kinase gene *OsPupK46–2* was identified [49–51]. Finally, *OsPupK46–2* was confirmed by analyzing the expression pattern of five candidate genes and named as *phosphorus-starvation tolerance 1* (*PSTOL1*) [49, 52]. In the present study, we integrated QTL mapping and trancriptomic profiling to identify candidate genes associated with root length responding to low-P stress. As a result, a QTL associated with root length was identified on chromosome 6H (Fig. 6). Previously, QTLs associated with grain yield and thousand grain weight under P treatments were reported on the same chromosome, but in the different regions in barley [42], indicating the QTL is a novel one associated with root length under low-P stress. In the identified region, 22 candidate genes were detected responding to low-P stress, including sugar transporter, isoflavone reductase, nitrate reductase, expansin B, MYB domain protein and a cluster of high affinity nitrate transporters (Fig. 6). Interestingly, the gene *HORVU6Hr1G002410*, encoding an isoflavone reductase (IFR), was up-regulated by the folds of 6.9 in L138 and 2.3 in L73 under low-P stress. In contrast, *HORVU6Hr1G003300* encoding a nitrate reductase 1 (NIA) was down-regulated by 2.8 folds in L138 and 7.8 folds in L73 when exposed to low-P stress.

IFRs are the enzymes unique to plant kingdom, involved in the biosynthetic pathway of isoflavonoid phytoalexin and have a crucial role in plant response to various biotic and abiotic stresses [53]. In kidney bean, the decrease of IFR expression level could affect the elongation of lateral root and the number of nodules [54]. In rice, IFR-like protein (IRL) may behave as an antioxidant in response to reactive oxygen species (ROS) during root development [55]. Thus, the identification of *IFR* as a candidate gene associated with root length indicates its potential role in plastic response to low-P stress in barley.

Nitrate reductase is well known for its role in nitrate reduction, which is critical for N uptake and utilization in the most plants. It was reported that phosphate application could increase the nitrate reductase activity [56], indicating more nitrogen was stored in the form of nitrate rather than nitrite under low-P condition. Some studies demonstrated that plants could be stimulated by localized application of nutrients. For instance, localized application of phosphates plus ammonium could enhance P uptake through stimulating root proliferation and rhizosphere acidification in a calcareous soil [57]; local applications of nitrate stimulates the growth of lateral root via reducing shoot-to-root auxin transport and decreasing auxin concentration in roots to a suitable level [58]. Thus, the identified nitrate reductase might contribute to local nitrate accumulation and further stimulate lateral growth for low-P tolerance. In this aspect, numerous DEGs encoding auxin responsive factors and auxin efflux carriers from transcriptomic profiling could provide the evidence for the assumed pathway (Fig. 5). In addition to NIA, a cluster of high affinity nitrate transporters were identified in the QTL region under low-P stress. On the other hand, nitrate-stimulated degradation of SPX4 activates a coordinated release of PHR and NLP which are TFs involved in phosphate and nitrate signalling pathways, respectively [59]. In the present study, we found three SPX genes were continuously induced by P-starvation, and numerous genes encoding NRT1 were responsive as well. Therefore, it may be suggested that nitrate signal might be involved in the response of lateral growth to low-P stress via regulating auxin and down-stream ARFs in barley (Additional file 4: Figure S4).

### Pi transport and P efficiency

P efficiency refers to the ability of plants to produce biomass or yield under certain available P conditions, and includes the aspects of P uptake efficiency and P utilization efficiency [60, 61]. In addition to root plasticity, improving the ability of Pi recycling within plants is also a strategy for improving P efficiency. In a Pi-depleted medium, the vacuole of plants acts as a storage pool and 85–95% of the total P in cell is located in vacuoles in the form of Pi [8]. Under P deficiency, a mass of Pi is transferred into the cytosol and chloroplasts in leaves, and plants may replace phospholipids by galactolipids or sulpholipids [62, 63]. Thus, the translocation of Pi to photosynthetic tissues is also considered to be an adaption of plants to low-P stress. In this study, a low-P tolerant barley line L138 had little change in biomass when shoot P concentration decreased from 10.8 into 1.3 mg P/g DW, but biomass of a sensitive line L73 was reduced by about 20% when shoot P concentration decreased from 9.8 to 1.4 mg P/g DW (Table 1). L73 and L138 had the similar shoot P concentration under low-P stress, but differed greatly in SDW, indicating that L73 had lower P-use efficiency than L138. Simultaneously, most DEGs encoding transporters involved in Pi transport within plants were induced by low-P stress, with L138 having higher change folds than L73 (Fig. 5a). The difference between L138 and L73 in the expression levels of three *PHO1* genes, encoding the endomembrane transporters responsible for Pi transport from root to shoot were more than 2 folds under low-P stress. In addition, the difference in expression levels of SPX-MFS family members were observed between the two barley lines, indicating that the active Pi efflux from vacuoles to cytoplasm might contribute to the low-P tolerance in L138 (Additional file 4: Figure S4).

In the highly regulated system in response to P starvation, sugar and numerous transcriptional factors such as WRKY, MYB and SPX3 families were involved in Pi translocation and homeostasis [27]. In the present study, we found a cluster of DEGs encoding sugar transporters were suppressed by low-P stress in L73 (Fig. 5c). Furthermore, most of the TFs DEGs in L138 were up-regulated, whereas, more than 60% of TFs DEGs were down-regulated in L73, indicating the different expression and regulation patterns between the two lines responding to P starvation. However, the proteins responsible for Pi transport within cells and the underlying molecular regulatory mechanisms in barley remain elusive. Currently it may be concluded that barley used the two strategies of root plasticity and Pi transport to develop the low-P tolerance.

## Conclusion

We identified a QTL associated with root length change in response to low-P stress and proposed candidate genes contributing to P efficiency in barley by integrating QTL mapping and transcriptomic profiling. The results demonstrated that the plasticity of root system contributes to the differences between barley genotypes in tolerance to low-P availability. L138, a DH line with high low-P tolerance is characterized by enhanced growth of lateral root and increased root-shoot transport of Pi under low-P condition. The candidate genes associated with low-P tolerance, encoding isoflavone reductase, nitrate reductase for lateral growth and the DEGs encoding PHO1, SPX-MFS for P mobility within plants were identified for their contribution to the low-P tolerance in L138 which is derived from a cross of Tibetan wild and cultivated barley.

## Methods

### Plant materials and growth condition

A barley double haploid (DH) population with 103 lines derived from a cross between Tibetan wild barley X26 and ZD9 was used for mapping and QTL analyses. The DH population was developed through microspore (anther) culture, and 2485 SNPs markers had been obtained. X26, a Tibetan wild barley accession showed higher adaption to abiotic stresses [64], and ZD9 is a cultivar planted locally. The seeds of X26 and ZD9 were obtained from Huazhong Agricultural University and Zhejiang University, China, respectively.

The seeds of all the DH lines were sterilized with 3%H_2_O_2_ for 30 min and rinsed with running water three times, thereafter transferred into sand bed. After germination, 7-day seedlings were transplanted into a hydroponic solution prepared in distilled water containing 200 μM K_2_SO_4_, 300 μM MgSO_4_·7H_2_O, 100 μM NaCl, 300 μM Mg(NO_3_)_2_·6H_2_O, 900 μM Ca(NO_3_)_2_·4H_2_O, 600 μM KNO_3_, 50 μM Fe(III)-EDTA-Na, 0.8 μM Na_2_MoO_4_·2H_2_O, 0.7 μM ZnCl2, 0.8 μM CuSO_4_·5H_2_O, 2 μM H_2_BO_3_ with pH 6.0. In the first week, the plants were grown in the solution containing 50 μM KH_2_PO_4_. In the following three weeks, plants were split into two regimes, 5 μM KH_2_PO_4_ for low-P treatment and 50 μM KH_2_PO_4_ for control. In an experiment of screening the two extreme lines, low-P starvation was conducted for 7 days (shorter duration) and for 14 days (longer duration). Plants were grown in a greenhouse with natural light, and a temperature of 20/15 °C day/night at Zijingang Campus, Zhejiang University, Hangzhou, China.

### Root and nutrient analyses

The fresh roots were collected and separated into seminal roots and crown roots. Seminal roots emerge from scutellar node and crown roots emerge from coleoptilar or tillering nodes. Thus mesocotyl was used to separate seminal roots and crown roots. All roots consist of axile roots and lateral roots, which are initiated from the division of pericycle and endodermis cells. All the roots were scanned by EPSON (v.700), and thereby analyzed using WinRHIZO (version 5.0). The length of total roots was calculated from the photos obtained by a scanner, and the length of axile rootswas measured by ruler. Additionally, the harvested plants were rinsed with distilled water and separated into roots and shoots for dry weight and nutrient analyses. After being dried in an oven at 75 °C for 48 h, the samples were digested in a mixture of concentrated nitric and perchloric acids. P concentration in the diluted solution was determined using an inductively coupled plasma-optical emission spectrometer (ICP-OES) (iCAP 6000 series, Thermo Fisher scientific, USA) according to the equipment operation manual.

### RNA isolation, library construction, sequencing and data processing

The roots of the two DH lines L138 and L73, which showed the significant difference in low-P tolerance was sampled at 7 days and 14 days after low-P treatment for transcriptomic profiling with three biological replicates for each sample. Total RNA was isolated according to the manual of miRNeasy mini kit (QIAGEN, Germany). RNA abundances and purity was determined by Agilent 2100 Bioanalyzer (Agilent Tecnologies, Santa Clara, CA, USA) and NanoDrop to meet the requirements. Magnetic beads with Oligo (dT) were used to isolate mRNA from total RNA. After fragmentation, cDNA was synthesized using the mRNA fragments as templates. Short fragments were purified and resolved for end reparation and single nucleotide adenine addition. Connected with adapters, DNA fragments were selectively amplified and enriched. During the quality control steps, Agilent 2100 Bioanaylzer (Agilent Technologies, Santa Clara CA, USA) and Real-Time PCR System (Applied Biosystems, Foster City, CA, USA) were used in quantification and qualification of the 24 sample libraries. Afterwards, PCR products were loaded into Illumina HiSeq™ 2000 (Illumina) for sequencing. The raw reads were trimmed by removing empty reads, adaptor sequences and low quality bases at the 3′ end to obtain the clean data. All the qualified reads were mapped to the barley reference genome and the reference gene set (IPK, 160517_Hv_IBSC_PGSB_r1_CDS_HighConf_REPR_annotation.fasta).

### Identification of the differentially expression genes and quantitative RT-PCR analyses

Gene expression levels were calculated using FPKM (fragments per kilo-base of exon per million fragments mapped reads) method [65]. The difference in expression between control and treatment was analyzed by the DESeq R package (1.10.1) [66]. Differentially expressed genes (DEGs) were required to have ≥2-fold change and *p* ≤ 0.05. The isolated RNA for transcriptomic profiling was used for quantitative RT- PCR assays to confirm the reliability for RNA-seq data. Total RNA was used as a template for reverse transcription after being treated with DNase. First strand cDNA was synthesized with oligo dT and Random 6 mers in a 20 μl reaction. Quantitative RT- PCR was performed on a CFX96 system machine (Bio-Rad, USA) as described by Zeng et al. (2014). The relative expression was calculated according to the comparative CT method [67] (Schmittgen and Livak, 2008). To normalize the gene expression, the amplification of *HvACTIN* sequence was used as housekeeping reference. The gene specific primers were designed using primer-blast (http://www.ncbi.nlm.nih.gov/tools/primer-blast/). All the primers were listed in Additional file 5: Table S1.

### Statistical analyses

The significance of difference between the barley genotypes or treatments in physiological traits was examined using SPSS software, followed by the Tukey test. The difference at *P* < 0.05 and 0.01 is thought as significant and highly significant, respectively. For the target trait, the average phenotypic of six replicates for each line was calculated for further QTL mapping. The genetic linkage map of X26/ZD9 DH population was constructed using 2485 SNP markers by software JoinMap 4 and QTLs were analyzed using software MapQTL 6.0. Interval mapping (IM) was performed firstly, and then the closest marker with highest Logarithm of the odds (LOD) score was set as a cofactor to test the multiple QTL model (MQM). A threshold LOD>2 was used to confirm the presence of a QTL in the IM and MQM analyses [68].

## Additional files

Additional file 1: **Figure S1.** Quantitative real-time PCR validation of 5 differentially expressed genes (DEGs) detected in L138 and L73 with low-P stress. Transcript levels of (a) PHO1-3, (b) ARF2, (c) SPX-MFS1, (d) SPX-MFS2, (e) sugar transporter 7 and the corresponding expression data of RNA-Seq are displayed by the columns and lines, respectively. (f) Comparison between the relative expressions (fold changes) obtained from RNA-Seq data and qPCR. (PPTX 399 kb)

Additional file 2: **Figure S2.** Hierarchical cluster (a) and gene functional categories analyses (b) of low-P tolerance related DEGs. (PPTX 54 kb)

Additional file 3: **Figure S3.** Hierarchical cluster analyses of nitrate transcporter1/peptide transporter (NRT1/PTR) identified in differentially expressed genes (DEGs). (PPTX 491 kb)

Additional file 4: **Figure S4.** The hypothetic pathways underlying low-P tolerant mechanisms in L138. ABA, Abscisic Acid; ARF, Auxin Responsefactor; LPR, Low Phosphate Root; PDR, Phosphate Deficiency Response; PHF, Phosphate Transporter Traffic Facilitator; PHO, Phosphate; PHR, Phosphate Starvation Response; PHT, Phosphate Transporter; PstB, Phosphate Import ATP-Binding Protein; SCR, Scarecrow; SPX, SYG1/PHO81/XPR1; SPX-MFS, SYG1/PHO81/XPR1-Major Facilitator Superfamily; TIR, Transport Inhibitor Response (PPTX 25 kb)

Additional file 5: **Table S1.** Primes used in quantitative Real-time PCR. (PPTX 22 kb)

## Data Availability

The datasets generated and analyzed during the current study, and the plant materials used in the presenting study are available from the corresponding author on reasonable request.

## Abbreviations

ARF2: Auxin response factor 2
ARP6: Actin-related protein 6
bHLH: Basic helix-loop-helix protein
DEG: Differentially expressed genes
EXS: ERD1/XPR1/SYG1
FPKM: Fragments per kilo-base of exon per million fragments mapped reads
IFR: Isoflavone reductase
MFS: Major facilitator superfamily
MYB: Myeloblastosis
NIA: Nitrate reductase 1
NLP: NIN-like protein
NRT1/ PTR (NPF): Nitrate or di/tri-peptide transporters
PHF1: Phosphate transporter traffic facilitator1
PHO1: Phosphate1
PHR1: Phosphate starvation response 1
PHT: Phosphate transporter
PSTOL1: Phosphorus-starvation tolerance 1
Pup1: Phosphorus uptake 1
RDW: Root dry weight
RL: Root length
SDW: Shoot dry weight
SPX: SYG1/Pho81/XPR1
ZAT: Zinc finger of Arabidopsis
