# Supplementary figures and images for: Root plasticity and Pi recycling within plants contribute to low-P tolerance in Tibetan wild barley
Source: BMC Plant Biol. 2019 Aug 5;19:341. doi: 10.1186/s12870-019-1949-x (PMC6683381; doi:10.1186/s12870-019-1949-x)

## Slide 1
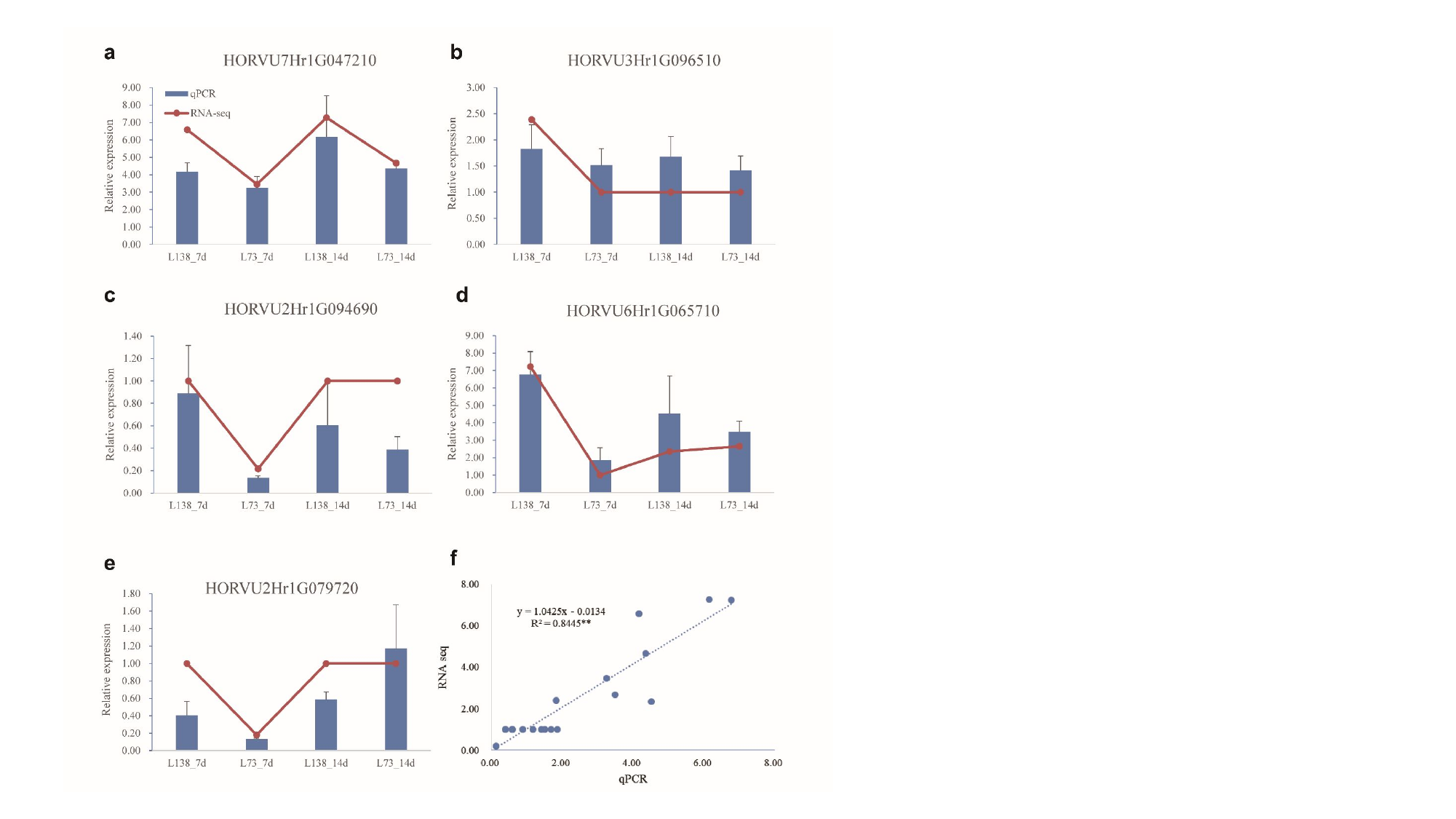

Supplement: Supplementary file 1 — Figure S1. Quantitative real-time PCR validation of 5 differentially expressed genes (DEGs) detected in L138 and L73 with low-P stress. Transcript levels of (a) PHO1-3, (b) ARF2, (c) SPX-MFS1, (d) SPX-MFS2, (e) sugar transporter 7 and the corresponding expression data of RNA-Seq are displayed by the columns and lines, respectively. (f) Comparison between the relative expressions (fold changes) obtained from RNA-Seq data and qPCR. (PPTX 399 kb) [file 12870_2019_1949_MOESM1_ESM.pptx]

## Slide 1
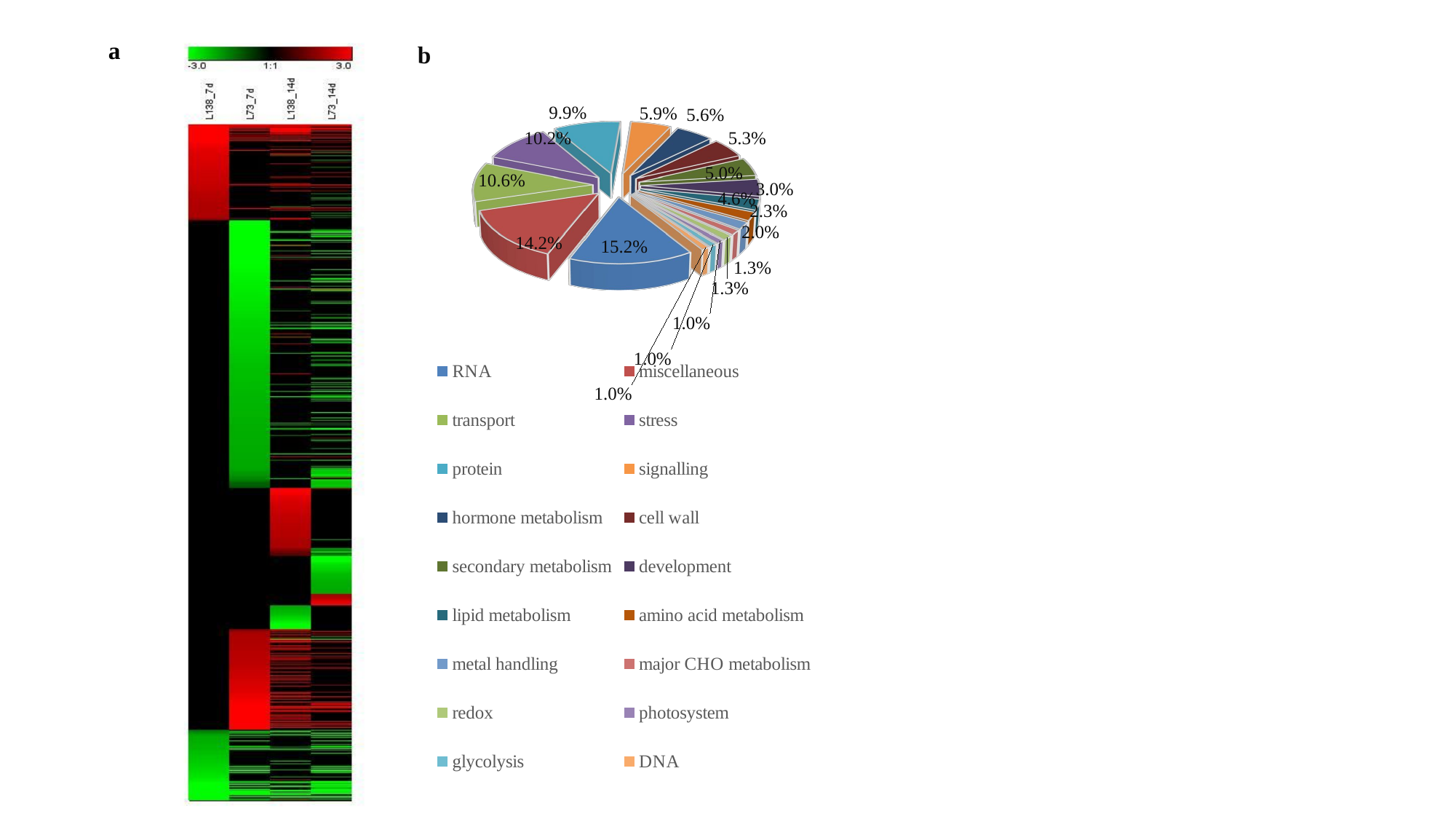

[unsupported chart]
a

Supplement: Supplementary file 2 — Figure S2. Hierarchical cluster (a) and gene functional categories analyses (b) of low-P tolerance related DEGs. (PPTX 54 kb) [file 12870_2019_1949_MOESM2_ESM.pptx]
