# Supplementary material for: Root plasticity and Pi recycling within plants contribute to low-P tolerance in Tibetan wild barley
Source: BMC Plant Biol. 2019 Aug 5;19:341. doi: 10.1186/s12870-019-1949-x (PMC6683381; doi:10.1186/s12870-019-1949-x)
Supplement: Supplementary file 4 — Figure S4. The hypothetic pathways underlying low-P tolerant mechanisms in L138. ABA, Abscisic Acid; ARF, Auxin Responsefactor; LPR, Low Phosphate Root; PDR, Phosphate Deficiency Response; PHF, Phosphate Transporter Traffic Facilitator; PHO, Phosphate; PHR, Phosphate Starvation Response; PHT, Phosphate Transporter; PstB, Phosphate Import ATP-Binding Protein; SCR, Scarecrow; SPX, SYG1/PHO81/XPR1; SPX-MFS, SYG1/PHO81/XPR1-Major Facilitator Superfamily; TIR, Transport Inhibitor Response (PPTX 25 kb) [file 12870_2019_1949_MOESM4_ESM.pptx]

## Slide 1
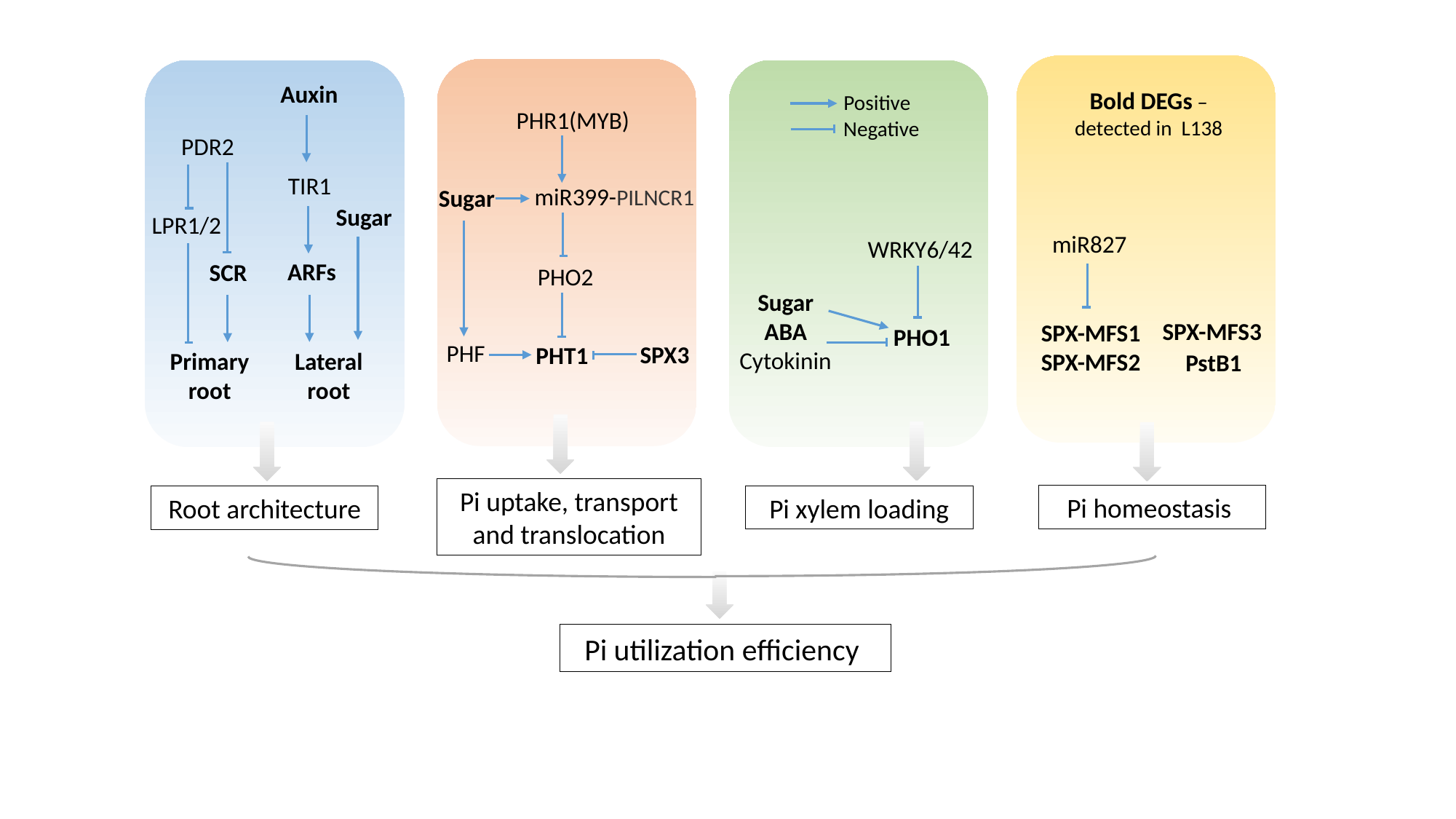

miR827
SPX-MFS3
SPX-MFS1
SPX-MFS2
Pi homeostasis
PHR1(MYB)
miR399-PILNCR1
Sugar
PHO2
PHF
SPX3
PHT1
Pi uptake, transport and translocation
WRKY6/42
Sugar
ABA
Cytokinin
PHO1
Pi xylem loading
Auxin
PDR2
TIR1
Sugar
LPR1/2
ARFs
SCR
Lateral root
Primary root
Root architecture
Bold DEGs –
detected in L138
Positive
Negative
PstB1
Pi utilization efficiency
