# Supplementary material for: Root plasticity and Pi recycling within plants contribute to low-P tolerance in Tibetan wild barley
Source: BMC Plant Biol. 2019 Aug 5;19:341. doi: 10.1186/s12870-019-1949-x (PMC6683381; doi:10.1186/s12870-019-1949-x)
Supplement: Supplementary file 5 — Table S1. Primes used in quantitative Real-time PCR. (PPTX 22 kb) [file 12870_2019_1949_MOESM5_ESM.pptx]

## Slide 1
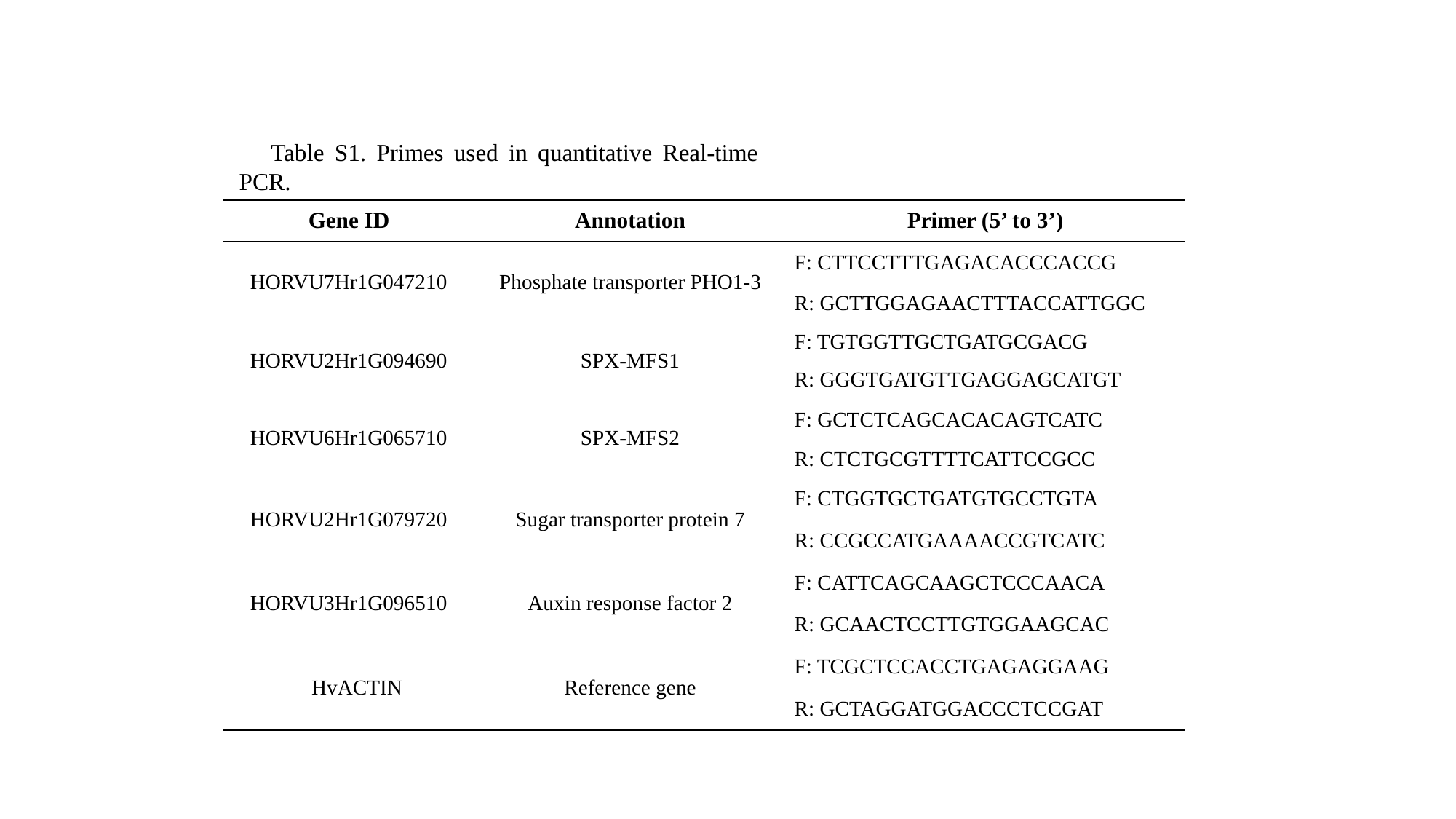

Table S1. Primes used in quantitative Real-time PCR.
| Gene ID | Annotation | Primer (5’ to 3’) |
| --- | --- | --- |
| HORVU7Hr1G047210 | Phosphate transporter PHO1-3 | F: CTTCCTTTGAGACACCCACCG |
| | | R: GCTTGGAGAACTTTACCATTGGC |
| HORVU2Hr1G094690 | SPX-MFS1 | F: TGTGGTTGCTGATGCGACG |
| | | R: GGGTGATGTTGAGGAGCATGT |
| HORVU6Hr1G065710 | SPX-MFS2 | F: GCTCTCAGCACACAGTCATC |
| | | R: CTCTGCGTTTTCATTCCGCC |
| HORVU2Hr1G079720 | Sugar transporter protein 7 | F: CTGGTGCTGATGTGCCTGTA |
| | | R: CCGCCATGAAAACCGTCATC |
| HORVU3Hr1G096510 | Auxin response factor 2 | F: CATTCAGCAAGCTCCCAACA |
| | | R: GCAACTCCTTGTGGAAGCAC |
| HvACTIN | Reference gene | F: TCGCTCCACCTGAGAGGAAG |
| | | R: GCTAGGATGGACCCTCCGAT |
